# Evidences of neurodegenerative processes in patients with late-onset schizophrenia and cognitive impairment

**DOI:** 10.1192/j.eurpsy.2022.1667

**Published:** 2022-09-01

**Authors:** V. Pochueva, M. Savina, V. Sheshenin, N. Cherkasov

**Affiliations:** FSBSI “Mental Health Research Centre”, Geriatric Psychiatry, Moscow, Russian Federation

**Keywords:** old age, late-onset schizophrenia, neuridegeneration, risk factors

## Abstract

**Introduction:**

The large proportion of patients with late-onset schizophrenia (LoS) has cognitive impairment. We hypothesized that this group of patients could have more risk factors associated with neurodegeneration.

**Objectives:**

The aimed to compare various clinical and risk factors in LoS patients with low and relatively preserved cognitive status.

**Methods:**

28 LoS patients (ICD-11) with duration of disease less than 10 years from a cohort of patients with late onset psychosis underwent clinical assessment (PANSS, HDRS-17), cognitive examination (MMSE, MoCA, FAB, verbal and symbolic memory, trail making test (part A, B)), structured interviewing on risk factors and CT. Hierarchical cluster analysis of cognitive test results was applied. Nonparametric statistic was used to compare control group (24 subjects with signs of psychosis or depression, age 58,1±10,8, 50% females) and patient`s groups.

**Results:**

Patients were divided on two clusters: Cluster 1 with lower cognitive functions (n=20, age 62,2, 94% of females) and Cluster 2 with preserved cognitive functions (n=8, age 56,8, 100% of females). Patients of Cluster 1 were older, had more negative symptoms, higher atrophy scores, higher rate of leukoaraiosis on CT and more history of mild brain injury than patients of Cluster 2 and controls. There was no group differences in age of manifestation, other PANSS scores, rates of social phobia and number of habitual anxiety reactions between clinical groups.

**Conclusions:**

LoS patients with cognitive deficiency had more factors associated with neurodegeneration, in particular history of mild brain injury.

**Disclosure:**

No significant relationships.

